# Weekly long-acting growth hormone versus daily growth hormone therapy on quality of life, efficacy, and safety in children with short stature: a systematic review and meta-analysis

**DOI:** 10.3389/fendo.2026.1907184

**Published:** 2026-09-02

**Authors:** Fan Liu, Linying Xiu, Wei Zhang, Chunhui Zang, Shuyu Wang

**Affiliations:** Department of Pediatrics, Bethune International Peace Hospital, Shijiazhuang, China

**Keywords:** children, daily growth hormone, long-acting growth hormone, meta-analysis, short stature

## Abstract

**Background:**

This meta-analysis aims to compare the QoL, efficacy, and safety of once-weekly LAGH versus daily GH in children with short stature.

**Methods:**

We systematically searched PubMed, Embase, the Cochrane Library, and Web of Science from inception to June 30, 2026, for studies comparing LAGH with daily GH in children with short stature. Outcomes of interest encompassed QoL, efficacy, and safety. Pooled analyses were conducted using a random-effects model. Treatment effects for continuous and dichotomous variables were expressed as weighted mean differences (WMDs) and risk ratios (RRs), respectively, each with 95% confidence intervals (CIs).

**Results:**

Thirty studies (27 cohorts) comprising 3,287 patients were included. Available QoL data from two major trials indicated comparable improvements between treatment groups, with a trend toward greater clinical benefit in psychosocial domains observed for a higher dose of Somapacitan. Regarding efficacy, no statistically significant differences were found between groups for height velocity (WMD: 0.26; 95% CI: -0.07 to 0.58; P = 0.124), height standard deviation score (SDS) (WMD: 0.04; 95% CI: -0.03 to 0.10; P = 0.285), or the ratio of bone age to chronological age (BA/CA) (WMD: 0.00; 95% CI: -0.01 to 0.01; P = 0.999). However, the LAGH group exhibited a significantly greater increase in insulin-like growth factor 1 (IGF-1) SDS compared to the daily GH group (WMD: 0.37; 95% CI: 0.13 to 0.62; P = 0.003). Subgroup analyses suggested that efficacy may be influenced by the specific LAGH formulation and baseline patient characteristics. The safety analysis revealed a comparable overall risk of adverse events between groups (RR: 1.02; 95% CI: 0.97 to 1.08; P = 0.468).

**Conclusion:**

Once-weekly LAGH demonstrates comparable efficacy and a similar overall safety profile to daily GH in children with short stature. By reducing injection frequency, LAGH offers the potential to enhance treatment convenience and adherence, which may translate into improved QoL. Further long-term studies are warranted to confirm these findings and to investigate formulation-specific effects.

## Introduction

Short stature is a common pediatric endocrine condition, typically defined as a height below the 3rd percentile or more than two standard deviations below the mean for children of the same age, sex, and ethnic background (1). Growth hormone deficiency (GHD) represents a well-established cause, with an estimated incidence ranging from 1 in 3,000 to 1 in 10,000 live births (2–4). Among children referred for evaluation of short stature, up to 70% exhibit no identifiable organic or endocrine abnormalities and are subsequently diagnosed with idiopathic short stature (ISS) (5). Both GHD and ISS may confer long-term physical and psychosocial risks, including impaired self-esteem, reduced social adaptation, and diminished quality of life (QoL) (6, 7).

Daily subcutaneous injections of recombinant human growth hormone (rhGH) have been the standard of care since the 1980s (8, 9). However, this regimen presents significant challenges for long-term adherence due to pain, lifestyle disruption, and psychological burden associated with frequent injections (10, 11). Suboptimal adherence correlates with diminished growth response, compromising overall therapeutic efficacy (12). To overcome these limitations, several once-weekly long-acting growth hormone (LAGH) formulations have been developed and approved for the treatment of GHD, with ongoing investigations in ISS populations (13). These formulations utilize various protein modification or drug delivery technologies to achieve sustained and stable release of GH. Their fundamental objective is to enhance treatment convenience and QoL by substantially reducing injection frequency—a strategy anticipated to improve long-term adherence and, consequently, clinical outcomes.

It is important to note, however, that once-weekly LAGH administration represents a fundamentally unphysiological approach compared to the physiological pulsatile secretion of endogenous GH or the daily exogenous GH replacement that more closely mimics the natural pattern. Endogenous GH is secreted in a pulsatile manner, predominantly during sleep, with peak levels followed by rapid clearance (14). Daily GH administration, while not perfectly replicating this pulsatility, at least maintains a daily rhythm that approximates the natural diurnal pattern. In contrast, once-weekly LAGH formulations produce sustained supraphysiological GH exposure over several days, with a markedly different pharmacokinetic and pharmacodynamic profile (15, 16). This sustained exposure pattern raises important questions regarding long-term safety, particularly concerning metabolic effects, tissue growth dynamics, and the potential for prolonged activation of the GH-IGF-1 axis (17). The potential implications of this unphysiological exposure profile warrant careful consideration and ongoing surveillance, especially as LAGH use expands beyond GHD into broader populations such as ISS.

Given the recent completion of multiple clinical studies comparing LAGH with daily GH in children with GHD or ISS, a systematic and quantitative synthesis of the available evidence is timely. Although several reviews have addressed this topic, a comprehensive meta-analysis encompassing all LAGH formulations, broadly evaluating their application across pediatric short stature populations, and providing an integrated assessment of QoL, efficacy, and safety is currently lacking (18, 19). Therefore, this study aims to systematically evaluate, via meta-analysis, the comparative therapeutic effects of once-weekly LAGH versus daily GH in the treatment of children with short stature.

## Materials and methods

### Search strategy and selection criteria

This systematic review and meta-analysis was conducted in accordance with the Preferred Reporting Items for Systematic Reviews and Meta-Analyses (PRISMA) statement (20). A comprehensive literature search was performed in the following electronic databases: PubMed, Embase, the Cochrane Central Register of Controlled Trials (CENTRAL), and the Web of Science Core Collection, from inception to June 30, 2026. We also searched ClinicalTrials.gov for grey literature and unpublished studies. The search strategy combined medical subject headings (MeSH) and free-text terms related to “long-acting growth hormone,” “once-weekly growth hormone,” “growth hormone deficiency,” “idiopathic short stature,” “children,” and “pediatric.” The full search strategy for each database is provided in **Supplementary File 1**. In addition, the reference lists of all included studies were manually screened to identify any further potentially eligible publications.

Two reviewers independently screened the titles, abstracts, and full texts of retrieved records against predefined inclusion and exclusion criteria. Disagreements were resolved through discussion or consultation with a third senior reviewer. Studies were selected according to the PICOS framework as follows: (1) Population: Children and adolescents (aged ≤ 18 years) with short stature requiring growth hormone therapy, including those diagnosed with GHD or ISS; (2) Intervention: Any once-weekly LAGH formulation, whether commercially available or under clinical investigation; (3) Comparator: Standard daily rhGH; (4) Outcomes: QoL, height velocity, height standard deviation score (SDS), bone age to chronological age ratio (BA/CA), insulin-like growth factor 1 (IGF-1) SDS, and adverse events; (5) Study design: Randomized controlled trials (RCTs) or observational studies (cohort or case-control studies).

### Data collection and quality assessment

Data were extracted independently by two reviewers using a pre-piloted, standardized data extraction form. The extracted information included: first author, publication year, study registration number, country, study design, sample size, participant demographics (age, sex, baseline anthropometric parameters), intervention and comparator details, follow-up duration, and outcome data (means, standard deviations, or event rates). Any discrepancies in data extraction were resolved through discussion or consultation with a third reviewer. Risk of bias for included RCTs was assessed using the Cochrane Risk of Bias tool, with each domain judged as “low risk,” “high risk,” or “unclear” (21). The methodological quality of observational studies was evaluated using the Newcastle-Ottawa Scale (NOS), with scores ranging from 0 to 9 (22). Quality assessments were performed independently by two reviewers, with disagreements resolved by consensus or arbitration by a third reviewer.

### Statistical analysis

For continuous outcomes, the treatment effect was expressed as the weighted mean difference (WMD) with its 95% confidence interval (CI). For dichotomous outcomes, the risk ratio (RR) with its 95% CI was used. Given the anticipated heterogeneity across studies, all pooled analyses were conducted using a random-effects model (23, 24). Heterogeneity was assessed using the I² statistic and Cochran’s Q test, with I² ≥ 50% or a P-value < 0.10 for the Q test considered indicative of significant heterogeneity (25, 26). To evaluate the robustness of the pooled estimates, sensitivity analyses were planned by sequentially omitting each individual study (27). Pre-specified subgroup analyses were conducted according to study design, sample size, age, proportion of male participants, type of LAGH formulation, and follow-up duration. Differences between subgroups were examined using interaction tests (28). Publication bias was assessed visually by inspecting funnel plots and quantitatively using Egger’s and Begg’s tests (29, 30). If publication bias was suspected, the trim-and-fill method was applied to adjust for its potential effect (31). All statistical tests were two-sided, and a P-value < 0.05 was considered statistically significant. All analyses were performed using STATA software, version 15.0 (StataCorp, College Station, TX, USA).

## Results

### Literature search and study selection

**Figure 1:**
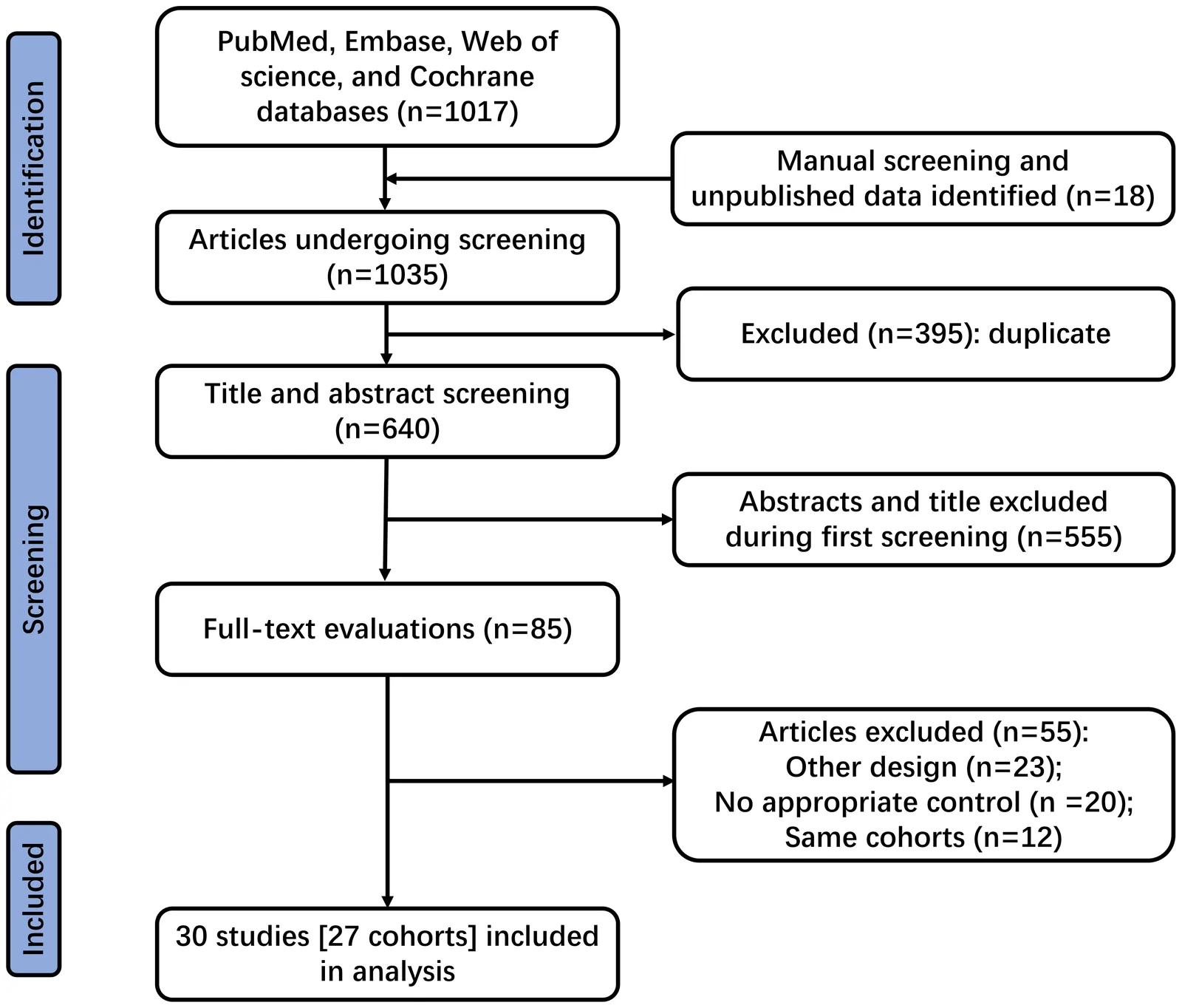
PRISMA Flowchart of study selection and inclusion process.

A total of 1017 records were identified through systematic database searches. After removing duplicates, 640 articles were screened based on titles and abstracts, of which 555 were excluded as clearly irrelevant. The full texts of the remaining 85 articles were assessed for eligibility, resulting in the exclusion of 55 articles for the following reasons: other study design (n=23), absence of a daily growth hormone control group (n=20), and duplicate reports from the same cohort (n=12). Ultimately, 27 cohorts reported across 30 studies met the predefined inclusion criteria and were included in this meta-analysis (32–61). The detailed study selection process and specific reasons for exclusion are presented in **Figure 1**.

### Trial characteristics

A total of 3,287 pediatric patients with short stature were enrolled across the included studies, comprising 1,878 in the LAGH group and 1,409 in the daily GH group. Twenty studies were designed as phase II to IV clinical trials, while the remaining seven cohorts were observational studies. Ten studies were multinational, and the remaining 17 were conducted in single countries, including China (n=13), South Korea (n=2), Japan (n=2), and Greece (n=1). Treatment duration ranged from 26 weeks to 3 years, with 18 studies having a core efficacy assessment period of 1 year (**Table 1**).

**Table 1:** The characteristics of identified trials and included individuals.

Trial	Study design	Country	Sample size (I/C)	Age (years) (I/C)	Male (%) (I/C)	Weight (kg) (I/C)	Height (cm) (I/C)	Intervention (LAGH)	Control (daily GH)	Follow-up (months)
Peter 2012 (32)	RCT	Europe	38 (26/12)	7.3 (7.3/7.3)	58.8 (59.0/58.3)	17.3 (16.6/18.1)	102.6 (102.2/103.0)	Eutropin Plus® (LB03002) (0.5/0.7 mg/kg/wk)	Genotropin® (0.03 mg/kg/d)	36.0
Hwang 2013 (33)	RCT	Korea	60 (30/30)	9.2 (9.1/9.3)	63.3 (66.7/60.0)	NA	NA	Eutropin Plus® (LB03002) (0.5 mg/kg/wk)	Eutropin® (0.03 mg/kg/d)	12.0
Khadilkar 2014 (34)	RCT	International	178 (91/87)	7.8 (7.8/7.8)	62.4 (61.5/63.2)	17.1 (17.0/17.2)	102.0 (102.2/101.8)	Eutropin Plus® (LB03002) (0.5 mg/kg/wk)	Genotropin® (0.03 mg/kg/d)	12.0
Luo 2017 (35)	RCT	China	65 (31/34); 343 (228/115)	11.0 (11.3/10.5); 11.5 (11.3/11.8)	73.2 (76.2/67.6); 81.6 (82.0/80.9)	21.2 (21.7/20.2); 23.7 (23.2/24.7)	112.3 (113.3/110.4); 117.3 (116.3/119.2)	Jintrolong® (0.2 mg/kg/wk)/Jintrolong® (0.2 mg/kg/wk)	Jintropin AQ® (0.0357 mg/kg/d)/ Jintropin AQ® (0.0357 mg/kg/d)	6.0
Chatelain 2017 (36)	RCT	Europe and Egypt	41 (28/13)	7.9 (8.0/7.7)	71.7 (70.0/76.9)	19.4 (19.3/19.6)	109.5 (110.2/107.4)	TransCon (ACP-001) (0.21/0.30 mg/kg/wk)	Genotropin® (0.03 mg/kg/d)	6.0
Zelinska 2017 (37)	RCT	Europe, USA, Israel	40 (29/11)	6.0 (6.0/5.7)	67.9 (66.7/72.7)	NA	NA	Somatrogon (MOD-4023) (0.48/0.66 mg/kg/wk)	Genotropin® (0.034 mg/kg/d)	12.0
Hwang 2018 (38)	RCT	Korea	42 (28/14)	5.8 (5.7/5.9)	69.0 (67.9/71.4)	17.7 (17.8/17.5)	105.8 (105.8/105.9)	Eutropin Plus® (LB03002) (0.5/0.7 mg/kg/wk)	rhGH (0.05 mg/kg/d)	24.0
Malievskiy 2018 (39)	RCT	Europe, Middle East and Korea	40 (26/14)	6.8 (6.8/6.9)	74.1 (77.5/64.3)	NA	NA	GX-H9 (1.2/2.4 mg/kg/wk)	Genotropin® (0.03 mg/kg/d)	12.0
Qiao 2019 (40)	Prospective cohort	China	98 (49/49)	5.8 (5.4/6.3)	60.2 (53.1/67.3)	NA	NA	Jintrolong® (0.2 mg/kg/wk)	Jintropin AQ® (0.43 mg/kg/d)	24.0
Savendahl 2020 (41, 42)	RCT	Europe, USA, Japan	28 (14/14)	5.9 (5.9/6.0)	59.6 (58.1/64.3)	14.6 (14.4/15.5)	97.2 (96.8/98.4)	Somapacitan (0.16 mg/kg/wk)	Norditropin® (0.034 mg/kg/d)	12.0
Sun 2021 (43)	RCT	China	363 (187/176)	7.9 (7.8/8.0)	70.8 (72.2/69.5)	20.6 (20.7/20.4)	113.2 (113.5/112.9)	Jintrolong® (0.2 mg/kg/wk)	Jintropin AQ® (0.0357 mg/kg/d)	6.0
Thornton 2021 (44)	RCT	Europe, USA, New Zeland	161 (105/56)	8.5 (8.5/8.5)	82.0 (81.9/82.1)	21.1 (21.0/21.2)	112.7 (112.9/112.2)	Lonapegsomatropin (TransCon) (0.24 mg/kg/wk)	Genotropin® (0.034 mg/kg/d)	12.0
Wan 2021 (45)	Retrospective cohort	China	50 (20/30)	5.8 (5.6-6.0)	58.0 (65.0/53.3)	17.9 (18.4/17.6)	106.3 (106.0/106.4)	Peg-rhGH (0.2 mg/kg/wk)	Daily rhGH (0.03 mg/kg/d)	12.0
Deal 2022 (46–48)	RCT	International	224 (109/115)	7.7 (7.8/7.6)	71.9 (75.2/68.7)	NA	NA	Somatrogon (MOD-4023) (0.66 mg/kg/wk)	Genotropin® (0.034 mg/kg/d)	12.0
Horikawa 2022 (49)	RCT	Japan	44 (22/22)	6.0 (5.3/6.8)	47.7 (40.9/54.5)	NA	NA	Somatrogon (MOD-4023) (0.66 mg/kg/wk)	Genotropin® (0.025 mg/kg/d)	12.0
Du 2022 (50)	RCT	China	46 (23/23)	9.4 (9.7/8.9)	50.7 (56.3/39.1)	25.3 (24.1/27.8)	119.8 (117.9/123.7)	Jintrolong® (0.20 mg/kg/wk)	Jintropin AQ® (0.04 mg/kg/d)	12.0
Gao 2022 (51)	Retrospective cohort	China	41 (27/14)	8.1 (7.9/8.9)	NA	22.6 (21.7/25.9)	110.7 (109.4/115.3)	Jintrolong® (0.2 mg/kg/wk)	Daily rhGH (0.054 mg/kg/d)	12.0
Mori 2024 (52)	RCT	Japan	30 (19/11)	5.8 (5.8/5.9)	66.7 (68.4/63.6)	15.6 (15.9/15.1)	99.0 (99.1/98.8)	Somapacitan (0.16 mg/kg/wk)	Norditropin® (0.034 mg/kg/d)	12.0
Xie 2024 (53)	Retrospective cohort	China	95 (47/48)	6.9 (6.6/7.3)	67.3 (61.7/72.9)	NA	NA	Jintrolong® (0.2 mg/kg/wk)	Jintropin AQ® (0.054 mg/kg/d)	24.0
Liang 2025 (54)	RCT	China	391 (261/130)	6.8 (6.7/7.0)	66.2 (65.9/66.9)	18.6 (18.5/18.8)	108.9 (108.4/109.9)	Pegpesen (0.14 mg/kg/wk)	rhGH (0.035 mg/kg/d)	12.0
Fu 2025 (55)	RCT	China	110 (74/36)	6.6 (6.6/6.5)	86.4 (87.8/83.3)	17.7 (17.7/17.7)	107.0 (107.4/106.3)	Somapacitan (0.16 mg/kg/wk)	Norditropin® (0.034 mg/kg/d)	12.0
Cai 2025 (56)	Prospective cohort	China	128 (64/64)	8.5 (8.6/8.4)	51.0 (48.4/56.3)	18.9 (18.9/18.8)	113.5 (113.4/113.7)	Jintrolong® (0.2 mg/kg/wk)	Jintropin AQ® (0.15 U/kg/d)	6.0
Abuzzahab 2026 (57)	RCT	International	88 (60/28)	2.5-11.0	40.9 (41.7/39.3)	17.4 (17.5/17.1)	105.7 (106.5/103.9)	Somapacitan (0.24 mg/kg/wk)	Norditropin® (0.050 mg/kg/d)	12.0
Ying 2026 (58)	RCT	China	153 (100/53)	7.4 (7.4/7.5)	86.3 (87.0/84.9)	19.9 (19.9/20.1)	111.1 (111.2/111.1)	Lonapegsomatropin (TransCon) (0.24 mg/kg/wk)	Norditropin® (0.034 mg/kg/d)	12.0
Guo 2026 (59)	Retrospective cohort	China	148 (85/63)	7.2 (6.9/7.5)	52.7 (50.6/55.6)	NA	NA	Peg-rhGH (0.25 mg/kg/wk)	Daily rhGH (0.04 mg/kg/d)	12.0
Stawerska 2026 (60)	RCT	International	212 (104/108)	8.8 (8.9/8.7)	70.8 (75.0/66.7)	NA	NA	Somatrogon (MOD-4023) (0.66 mg/kg/wk)	Norditropin® (0.034 mg/kg/d)	12.0
Moutafi 2026 (61)	Retrospective cohort	Greece	30 (15/15)	12.2 (12.4/12.0)	53.3 (46.7/60.0)	NA	NA	Somatrogon (MOD-4023) (0.66 mg/kg/wk)	rhGH (0.16-0.24 mg/kg/d)	18.0

Methodological quality of the included RCTs was assessed using the Cochrane Risk of Bias tool. Overall, the included studies exhibited a low to moderate risk of bias (**Supplementary File 2**). All trials clearly described their method of random sequence generation; however, allocation concealment was unclear in 11 studies. Due to the nature of the intervention, blinding of participants and personnel was challenging; therefore, blinding was primarily applied to outcome assessors. Additionally, seven studies were rated as having a high risk of bias due to incomplete outcome data, and three studies were judged to have an unclear risk regarding selective outcome reporting. The quality of the five included observational studies was assessed using the NOS, with one study receiving 9 stars, two study receiving 8 stars, and the remaining four studies receiving 7 stars (**Supplementary File 2**).

### QoL

Two study cohorts (reported in three publications) compared the impact of LAGH versus daily GH on QoL, including the REAL 3 trial (NCT02616562) (41, 42) and the OPKO Health trial (NCT02968004) (46–48). The REAL 3 study evaluated once-weekly Somapacitan (at three weekly doses) versus daily GH (Norditropin^®^) over 52 weeks using a disease-specific measure, the GHD-CIM ObsRO (observer-reported outcome). The OPKO Health study evaluated once-weekly Somatrogon versus daily GH (Genotropin^®^/somatropin) over 12 months using the generic QoLISSY questionnaire.

The primary conclusion from both trials was that LAGH (both Somapacitan and Somatrogon) and daily GH demonstrated comparable effectiveness in improving QoL in children with GHD, with no statistically significant differences observed. However, some noteworthy trends emerged. In the REAL 3 study, the change from baseline in the emotional wellbeing and social wellbeing domains, as well as the total score at week 52 for the highest Somapacitan dose (0.16 mg/kg/week), exceeded the minimal important difference compared to daily GH, suggesting a clinically relevant improvement favoring Somapacitan, although statistical significance was not reached. In the OPKO Health study, the self-reported QoLISSY-Child total score showed a numerically greater improvement with Somatrogon versus daily GH (mean change: 13.00 vs. 7.84), though statistical comparison was not reported, and the overall conclusion emphasized comparable improvements between groups.

Given that REAL 3 used a disease-specific instrument (GHD-CIM) while OPKO Health used a generic instrument (QoLISSY), a quantitative meta-analysis was not performed. Synthesizing the available evidence, once-weekly LAGH regimens demonstrated similar QoL benefits to daily GH over the studied period (52 weeks/12 months) in children with GHD. The higher dose of Somapacitan may suggest a trend toward more clinically meaningful improvement in emotional and social functioning. Differences in QoL assessment tools and respondent perspectives (child vs. parent) may influence interpretation, but the overall findings support LAGH as an effective treatment option for improving QoL, with the potential added convenience of a simplified weekly injection schedule.

### Height velocity

**Figure 2:**
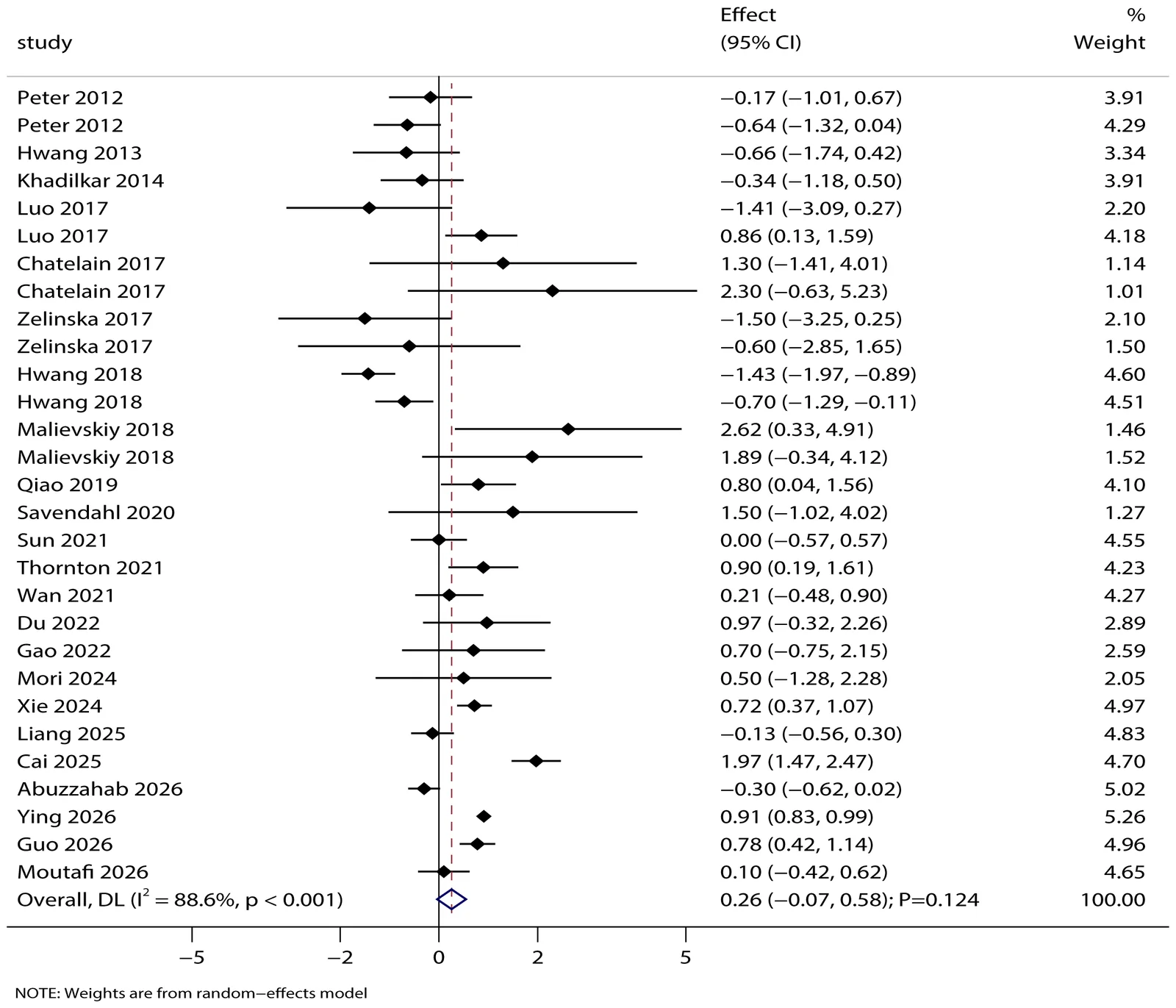
Forest plot of height velocity after treatment with long-acting growth hormone versus daily growth hormone.

A total of 23 studies reported post-treatment height velocity. Meta-analysis results indicated no statistically significant difference in height velocity between the LAGH group and the daily GH group (WMD: 0.26; 95% CI: -0.07 to 0.58; P = 0.124; **Figure 2**), with significant heterogeneity observed across studies (I² = 88.6%; P < 0.001). Sensitivity analysis confirmed that the difference in height velocity between the two groups remained stable and was not influenced by any single study (**Supplementary File 3**). Subgroup analysis revealed that when Eutropin Plus^®^ was used as the LAGH formulation, height velocity was significantly lower in the long-acting group. Conversely, in pooled cohort studies, studies with a sample size ≥ 100, a mean age ≥ 7.0 years, or when the proportion of male participants was ≥ 70.0%, or when Jintrolong^®^, TransCon, or GX-H9 was used as the LAGH formulation, height velocity was significantly higher in the long-acting group (**Table 2**). The difference in height velocity between the two groups was influenced by study design (P = 0.033), sample size (P = 0.034), mean age of patients (P = 0.013), and the type of LAGH used (P < 0.001). Reviewing the funnel plot could not rule out publication bias for height velocity. Although the Begg test indicated no significant publication bias (P = 0.138), whereas the Egger test suggested potential significant publication bias (P = 0.022). After adjusting using the trim and fill method, the conclusion was robustness and not affected (WMD: 0.20; 95% CI: -0.13 to 0.53; P = 0.230) (**Supplementary File 4**).

**Table 2:** Subgroup analyses for height velocity, height SDS, BA/CA, and IGF-1 SDS.

Outcomes	Variables	Subgroup	No of studies	MD and 95%CI	*P* value	*I^2^* (%)	Q statistic	Interaction test
Height velocity	Study design	RCT	16	0.06 (-0.39 to 0.51)	0.789	90.1	< 0.001	0.033
		Cohort	7	0.77 (0.30 to 1.24)	0.001	80.7	< 0.001	
	Sample size	≥ 100	8	0.65 (0.23 to 1.06)	0.002	87.8	< 0.001	0.034
		< 100	16	0.03 (-0.37 to 0.43)	0.888	75.6	< 0.001	
	Age (years)	≥ 7.0	13	0.43 (0.07 to 0.80)	0.021	82.5	< 0.001	0.013
		< 7.0	9	0.11 (-0.49 to 0.70)	0.724	83.2	< 0.001	
	Male (%)	≥ 70.0	6	0.72 (0.24 to 1.20)	0.003	61.6	0.008	0.107
		< 70.0	16	0.06 (-0.36 to 0.47)	0.790	87.3	< 0.001	
	LAGH	Eutropin Plus®	4	-0.72 (-1.12 to -0.33)	< 0.001	43.3	0.116	< 0.001
		Jintrolong®	7	0.71 (0.13 to 1.28)	0.016	80.3	< 0.001	
		TransCon	3	0.91 (0.83 to 0.99)	< 0.001	0.0	0.814	
		Somatrogon	1	-1.16 (-2.54 to 0.22)	0.099	0.0	0.536	
		GX-H9	1	2.24 (0.65 to 3.84)	0.006	0.0	0.655	
		Somapacitan	4	-0.09 (-0.47 to 0.30)	0.661	22.5	0.276	
		Pegpesen	3	0.30 (-0.32 to 0.92)	0.343	80.7	0.006	
	Follow-up (months)	≥ 12.0	19	0.15 (-0.21 to 0.50)	0.422	89.4	< 0.001	0.295
		< 12.0	4	0.74 (-0.32 to 1.80)	0.169	85.9	< 0.001	
Height SDS	Study design	RCT	16	0.02 (-0.03 to 0.07)	0.483	13.6	0.291	0.308
		Cohort	6	0.14 (-0.09 to 0.37)	0.231	75.5	0.001	
	Sample size	≥ 100	7	0.10 (0.00 to 0.20)	0.040	73.2	0.001	0.048
		< 100	12	-0.03 (-0.12 to 0.06)	0.505	15.7	0.269	
	Age (years)	≥ 7.0	10	0.07 (-0.03 to 0.17)	0.184	53.2	0.012	0.527
		< 7.0	7	0.00 (-0.10 to 0.10)	0.945	39.9	0.091	
	Male (%)	≥ 70.0	4	0.09 (-0.01 to 0.18)	0.073	50.5	0.089	0.398
		< 70.0	13	0.02 (-0.08 to 0.11)	0.738	47.8	0.013	
	LAGH	Eutropin Plus®	4	-0.10 (-0.21 to 0.02)	0.096	0.0	0.966	0.001
		Jintrolong®	5	0.03 (-0.03 to 0.08)	0.312	0.0	0.571	
		Somapacitan	4	-0.12 (-0.28 to 0.052)	0.161	0.0	0.470	
		TransCon	2	0.16 (0.07 to 0.25)	< 0.001	0.0	0.654	
		Pegpesen	3	0.29 (-0.13 to 0.71)	0.171	89.1	< 0.001	
	Follow-up (months)	≥ 12.0	16	0.04 (-0.04 to 0.12)	0.345	53.2	0.002	0.559
		< 12.0	2	0.01 (-0.05 to 0.07)	0.767	0.0	0.895	
BA/CA	Study design	RCT	8	-0.00 (-0.01 to 0.01)	0.974	0.0	0.559	0.792
		Cohort	1	0.01 (-0.06 to 0.08)	0.794	–	–	
	Sample size	≥ 100	4	0.00 (-0.02 to 0.02)	0.865	21.1	0.283	0.740
		< 100	5	-0.00 (-0.02 to 0.02)	0.770	0.0	0.689	
	Age (years)	≥ 7.0	6	0.00 (-0.01 to 0.01)	0.883	0.0	0.513	0.795
		< 7.0	2	-0.00 (-0.02 to 0.02)	0.721	5.3	0.348	
	Male (%)	≥ 70.0	3	-0.00 (-0.01 to 0.01)	0.750	0.0	0.551	0.564
		< 70.0	6	0.00 (-0.01 to 0.02)	0.631	0.0	0.505	
	LAGH	Eutropin Plus®	4	0.00 (-0.02 to 0.03)	0.731	17.2	0.302	0.827
		Jintrolong®	1	0.00 (-0.01 to 0.01)	1.000	–	–	
		TransCon	2	-0.02 (-0.05 to 0.02)	0.317	0.0	0.589	
		Pegpesen	1	0.01 (-0.06 to 0.08)	0.794	–	–	
		Somapacitan	1	0.03 (-0.08 to 0.14)	0.577	–	–	
	Follow-up (months)	≥ 12.0	8	0.00 (-0.02 to 0.02)	0.999	0.0	0.552	0.999
		< 12.0	1	0.00 (-0.01 to 0.01)	1.000	–	–	
IGF-1 SDS	Study design	RCT	11	0.46 (0.23 to 0.69)	< 0.001	63.6	< 0.001	0.626
		Cohort	4	0.25 (-0.58 to 1.08)	0.557	88.4	< 0.001	
	Sample size	≥ 100	6	0.61 (0.35 to 0.87)	< 0.001	76.7	< 0.001	0.068
		< 100	10	0.15 (-0.26 to 0.57)	0.465	68.5	< 0.001	
	Age (years)	≥ 7.0	7	0.59 (0.33 to 0.85)	< 0.001	70.5	0.001	0.216
		< 7.0	7	0.12 (-0.37 to 0.61)	0.622	77.8	< 0.001	
	Male (%)	≥ 70.0	5	0.58 (0.30 to 0.85)	< 0.001	78.6	< 0.001	0.128
		< 70.0	10	0.19 (-0.23 to 0.61)	0.376	69.4	< 0.001	
	LAGH	Eutropin Plus®	3	-0.00 (-0.73 to 0.72)	0.992	42.9	0.154	0.056
		Jintrolong®	4	0.17 (-0.22 to 0.56)	0.401	85.7	< 0.001	
		Somapacitan	5	0.38 (0.03 to 0.73)	0.032	0.0	0.908	
		TransCon	2	0.99 (0.48 to 1.50)	< 0.001	79.5	0.027	
		Pegpesen	1	0.70 (0.26 to 1.14)	0.002	–	–	
	Follow-up (months)	≥ 12.0	13	0.37 (-0.00 to 0.73)	0.051	80.0	< 0.001	0.878
		< 12.0	2	0.40 (0.27 to 0.53)	< 0.001	0.0	0.603	

### Height SDS

**Figure 3:**
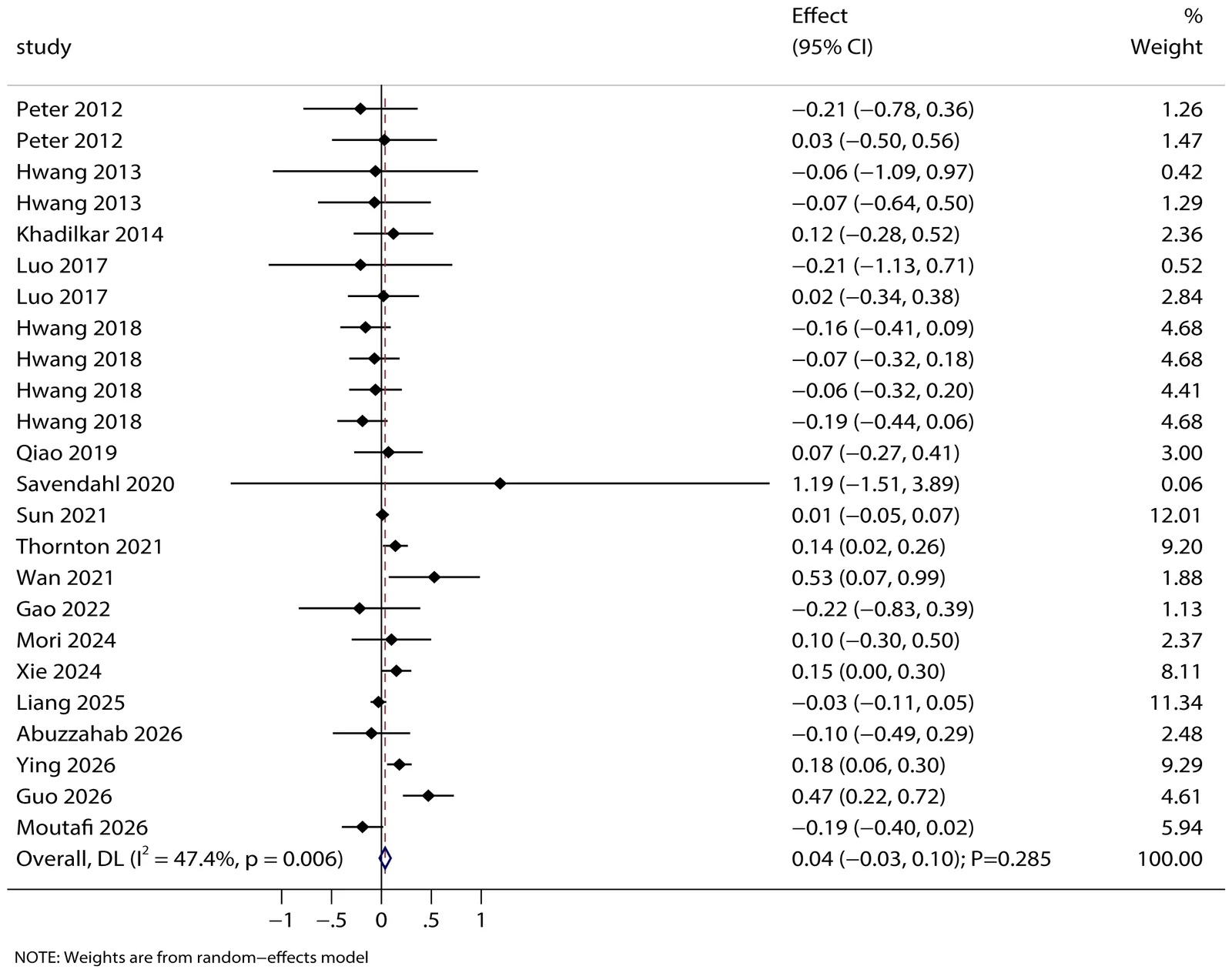
Forest plot of change in height standard deviation score after treatment with long-acting growth hormone versus daily growth hormone.

A total of 22 studies reported post-treatment height SDS. The pooled analysis indicated no significant difference in the improvement of height SDS between the two groups (WMD: 0.04; 95% CI: –0.03 to 0.10; P = 0.285; **Figure 3**), with moderate heterogeneity among studies (I² = 47.4%; P = 0.006). Sensitivity analysis showed that the difference in height SDS between the LAGH group and the daily GH group remained stable and was not influenced by any single study (**Supplementary File 3**). Subgroup analysis revealed that when TransCon was used as the long-acting formulation, or studies with a sample size ≥ 100, the improvement in height SDS was significantly greater in the LAGH group (**Table 2**). The observed difference in height SDS levels was influenced by sample size (P = 0.048), and the type of LAGH used (P = 0.001). Assessment for publication bias indicated no significant publication bias for height SDS (Egger’s test P = 0.886; Begg’s test P = 0.519) (**Supplementary File 4**).

### BA/CA

**Figure 4:**
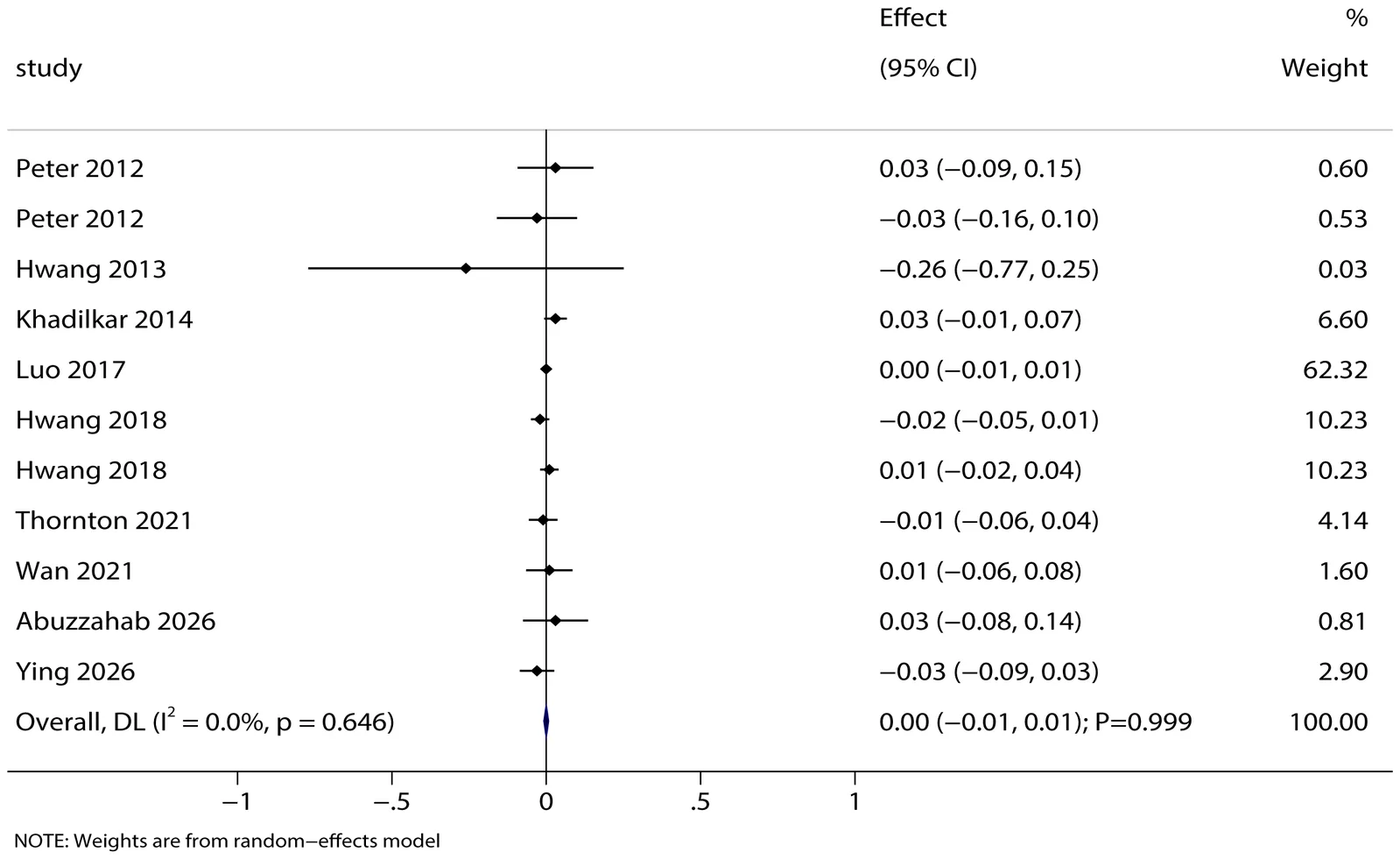
Forest plot of bone age/chronological age ratio after treatment with long-acting growth hormone versus daily growth hormone.

A total of 9 studies reported post-treatment BA/CA. Our analysis indicated no statistically significant difference in the effect on BA/CA between the LAGH group and the daily GH group (WMD: 0.00; 95% CI: –0.01 to 0.01; P = 0.999; **Figure 4**), with no significant heterogeneity among studies (I² = 0.0%; P = 0.646). Sensitivity analysis showed that the pooled results for BA/CA were stable and not influenced by any single study (**Supplementary File 3**). Across all subgroups, no significant differences in BA/CA were observed between the two treatment groups (**Table 2**). No significant publication bias was detected for BA/CA (Egger’s test P = 0.744; Begg’s test P = 0.696) (**Supplementary File 4**).

### IGF-1 SDS

**Figure 5:**
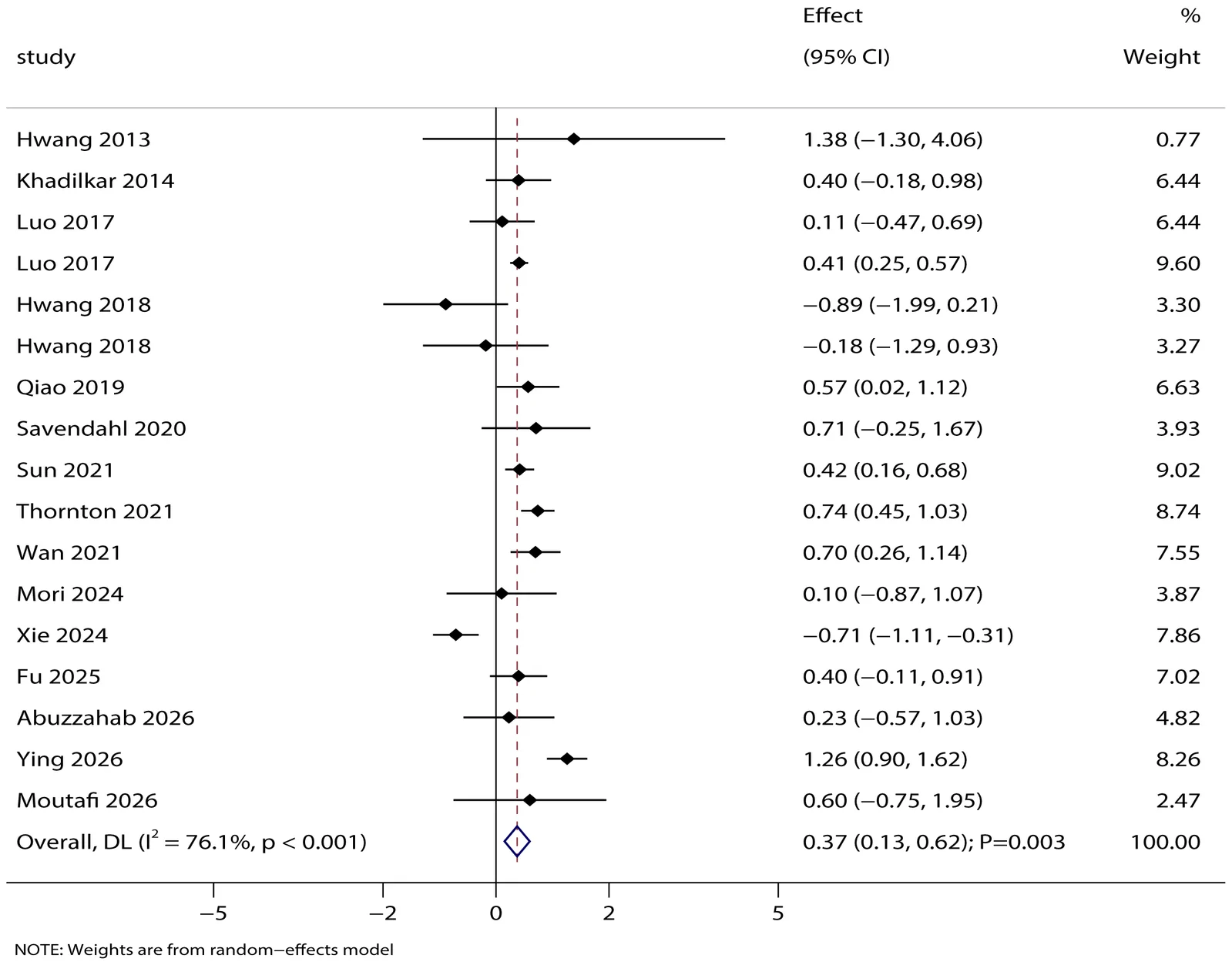
Forest plot of insulin-like growth factor-1 standard deviation score after treatment with long-acting growth hormone versus daily growth hormone.

A total of 15 studies reported post-treatment IGF-1 SDS. Our analysis indicated that the LAGH group was associated with a significantly greater increase in IGF-1 SDS compared to the daily GH group (WMD: 0.37; 95% CI: 0.13 to 0.62; P = 0.003; **Figure 5**), with significant heterogeneity observed among studies (I² = 76.1%; P < 0.001). Sensitivity analysis suggested that the pooled conclusion was robustness and not affected by any particular study (**Supplementary File 3**). Subgroup analysis revealed that in RCTs, studies with a sample size ≥ 100, a mean age ≥ 7.0 years, a male proportion ≥ 70.0%, or those using Somapacitan, TransCon or Pegpesen as the LAGH formulation, or with a follow-up duration < 12.0 months, the LAGH group showed a significantly greater increase in IGF-1 SDS compared to the daily GH group (**Table 2**). No significant publication bias was detected for IGF-1 SDS (Egger’s test P = 0.600; Begg’s test P = 0.537) (**Supplementary File 4**).

### Adverse events

**Figure 6:**
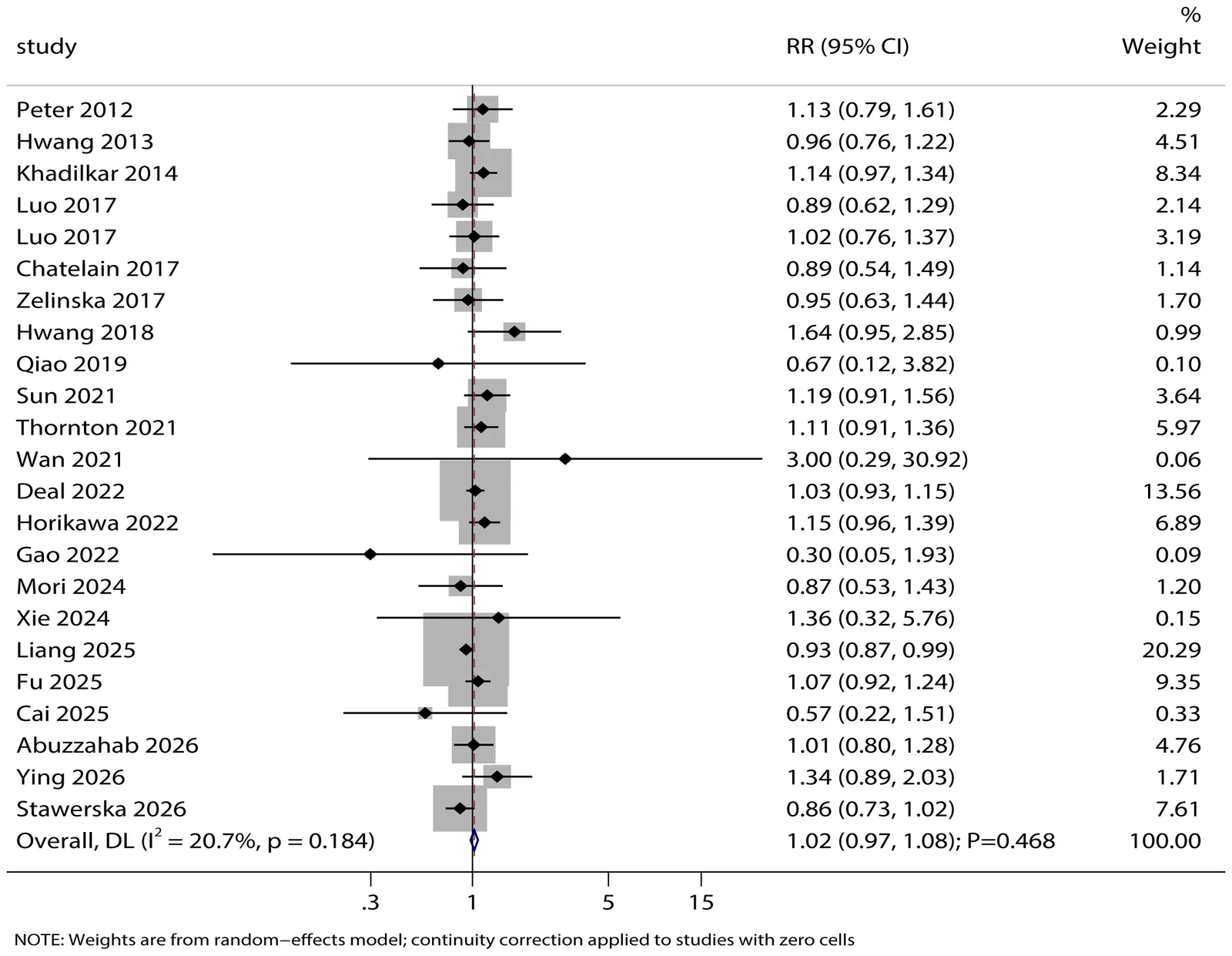
Forest plot of risk of treatment-related adverse events with long-acting growth hormone versus daily growth hormone.

A total of 22 studies reported treatment-related adverse events. Our analysis indicated no statistically significant difference in the risk of adverse events between the LAGH group and the daily GH group (RR: 1.02; 95% CI: 0.97–1.08; P = 0.468; **Figure 6**), with no significant heterogeneity among studies (I² = 20.7%; P = 0.184). Sensitivity analysis showed that the pooled conclusion for adverse events was not affected by individual study (**Supplementary File 3**). No significant publication bias was detected for adverse events (Egger’s test P = 0.248; Begg’s test P = 0.398) (**Supplementary File 4**).

## Discussion

This meta-analysis, comprising 27 cohorts from 30 studies with a total of 3,287 pediatric patients, provides a comprehensive synthesis of the comparative effects of once-weekly LAGH versus daily GH in children with short stature. The key findings can be summarized as follows. First, regarding QoL—a primary rationale for the development of long-acting formulations—the available evidence, although limited, indicates that LAGH yields improvements comparable to daily GH, with a potential trend toward greater clinical benefit in psychosocial domains observed with a higher dose of Somapacitan. Second, in terms of core efficacy outcomes, LAGH demonstrates non-inferiority to daily therapy in promoting linear growth, as evidenced by the absence of statistically significant differences in height velocity, height SDS, or bone maturation (BA/CA). Third, the overall safety profiles of the two regimens are largely similar, with no significant difference in the overall risk of adverse events. However, a notable finding was the significantly higher IGF-1 SDS observed in the LAGH group compared to the daily GH group. Collectively, these results support the therapeutic equivalence of once-weekly LAGH in terms of growth promotion and overall safety, while highlighting its potential added value in treatment convenience and identifying a differential effect on IGF-1 generation.

Several recent systematic reviews and meta-analyses have addressed the comparative effectiveness of LAGH versus daily GH in children. Bin-Abbas and Jabari (18) conducted a meta-analysis specifically focusing on pegylated recombinant human GH, reporting comparable efficacy and safety between once-weekly pegylated GH and daily GH, consistent with our overall findings. However, their analysis was restricted to a single LAGH class (pegylated formulations) and did not comprehensively evaluate QoL outcomes or provide detailed subgroup analyses across different LAGH technologies. Liu et al. (62) performed a network meta-analysis examining the comparative efficacy and safety of Jintrolong and other LAGH formulations specifically in East Asian populations with GHD. Their work provided valuable region-specific insights but was geographically limited and did not include comprehensive QoL assessment or analysis of BA/CA outcomes. Levaillant et al. (63) conducted a meta-analysis of RCTs focusing specifically on changes in body mass index (BMI) during LAGH treatment, providing important metabolic data but with a narrow scope limited to BMI outcomes and RCTs only. Basile et al. (64) provided a comprehensive narrative review of LAGH effectiveness and safety, offering broad contextual insights but without quantitative meta-analytic synthesis of efficacy outcomes across different LAGH formulations. Our meta-analysis extends and complements these previous works in several important ways. First, we provide the most comprehensive synthesis to date, incorporating 22 cohorts from 25 studies with 2,656 patients, encompassing all major LAGH formulations (Somapacitan, Somatrogon, TransCon, Jintrolong, GX-H9, LB03002, and Eutropin Plus) across both GHD and ISS populations. Second, we uniquely integrate QoL assessment alongside efficacy and safety outcomes, providing a more holistic evaluation of treatment value. Third, we provide detailed subgroup analyses examining the influence of specific LAGH formulations, study design, and patient characteristics on treatment outcomes. Fourth, we specifically address the concerning finding of elevated IGF-1 SDS with LAGH and discuss its implications in the context of the unphysiological GH exposure profile, a topic that has received limited attention in previous meta-analyses. Finally, we include both RCTs and real-world observational studies, enhancing the external validity and generalizability of our findings. These distinct features establish the novelty and clinical relevance of our work.

A critical yet underexplored dimension of this comparison pertains to QoL. Although limited to two major trials, the preliminary data are highly instructive. Both the REAL 3 (Somapacitan) (41, 42) and OPKO Health (Somatrogon) (46–48) studies concluded that LAGH and daily GH yielded comparable improvements in QoL over 52 weeks or 12 months in children with GHD, with no statistically significant differences between regimens. Notably, a clinically relevant trend favoring the higher dose of Somapacitan was observed in emotional and social functioning domains. Reducing the injection burden from approximately 365 to about 52 injections per year addresses a core challenge of daily GH therapy—treatment adherence—which is intrinsically linked to long-term growth outcomes and the psychosocial well-being of children and their families (10). This enhanced convenience represents a primary and compelling rationale for LAGH, with the potential to translate into improved sustained efficacy and enhanced patient experience, thereby constituting its most significant potential added value beyond biological equivalence.

Regarding core growth outcomes, this analysis demonstrates no statistically significant difference between once-weekly LAGH and daily GH in terms of height velocity, improvement in height SDS, or bone maturation (BA/CA). The consistency observed in height SDS, robustly supports the clinical equivalence of LAGH in promoting linear growth without adversely affecting skeletal maturation. This finding aligns with the pharmacological objective of LAGH formulations to provide sustained GH exposure that mimics physiological effects (61, 65). However, the significant heterogeneity in height velocity warrants careful interpretation. Subgroup analyses indicate that efficacy may be influenced by specific LAGH formulations. For instance, formulations such as Jintrolong^®^, TransCon, or GX-H9 were associated with significantly greater height velocity in the LAGH group, whereas Eutropin Plus^®^ was associated with lower velocity compared to daily GH. Similarly, a significantly greater improvement in height SDS was observed with the TransCon formulation. These findings underscore that LAGH products are not interchangeable as a class; their distinct molecular designs and resulting clinical profiles must be considered individually in clinical practice (66, 67).

A distinct finding of this analysis is the significantly higher IGF-1 SDS in the LAGH group compared to the daily GH group. This effect was particularly evident in predefined subgroups, including RCTs, larger studies (sample size ≥ 100), a mean age ≥ 7.0 years, a male proportion ≥ 70.0%, studies using Somapacitan, TransCon or Pegpeson formulations, and those with shorter follow-up (< 12 months). This elevated IGF-1 response warrants serious consideration in the context of the unphysiological sustained GH exposure profile of LAGH formulations. Unlike daily GH, which provides intermittent stimulation of the GH-IGF-1 axis and allows for periods of receptor desensitization and metabolic recovery, once-weekly LAGH generates continuous supraphysiological GH levels that may lead to persistent activation of the IGF-1 pathway (11, 17). This sustained elevation is particularly concerning given the well-established role of the GH-IGF-1 axis in cell proliferation and anti-apoptosis (68). Epidemiological and clinical studies have consistently linked elevated IGF-1 levels to increased risks of various malignancies, including breast, colorectal, and prostate cancers (69–71). In the pediatric population, the long-term implications of sustained IGF-1 elevation remain unknown, as the growth-promoting effects of GH therapy are typically evaluated over relatively short time horizons (1–3 years). However, the potential for increased neoplasm incidence, particularly in patients with pre-existing risk factors, cannot be dismissed (68). Of critical importance, LAGH treatment is currently not approved for cancer survivor patients or those with active malignancies, reflecting the theoretical concerns regarding IGF-1-mediated tumorigenesis (72). While short-term data have not revealed related clinical sequelae, and current evidence suggests comparable overall safety profiles between LAGH and daily GH in the short term, the elevated IGF-1 SDS observed in this meta-analysis highlights the urgent need for long-term pharmacovigilance. We recommend systematic long-term monitoring of IGF-1 levels, metabolic parameters (particularly glucose metabolism), and malignancy surveillance in children receiving LAGH, especially with formulations associated with higher IGF-1 responses (73). Future studies should incorporate extended follow-up periods (≥5–10 years) to adequately assess the risk-benefit profile of LAGH in the pediatric population.

The principal strengths of this study include its rigorous adherence to PRISMA guidelines, a comprehensive search strategy, and the inclusion of diverse LAGH formulations across both GHD and ISS indications, enhancing the generalizability of the findings. Several limitations must be acknowledged. First, methodological constraints in some included studies may introduce bias. Second, the predominance of one-year follow-up data precludes definitive conclusions regarding long-term efficacy and safety. Third, there is a notable scarcity of patient-reported outcomes and objective adherence metrics beyond the limited QoL data. Finally, although subgroup analyses yielded insightful hypotheses regarding the impact of LAGH type and population characteristics, they are based on study-level aggregate data, and some subgroups contained few studies, necessitating cautious interpretation.

## Conclusion

In summary, this meta-analysis demonstrates that once-weekly LAGH is comparable to daily GH in key efficacy and safety outcomes for children with short stature. Its potential to significantly reduce injection frequency may confer meaningful benefits in treatment convenience, adherence, and QoL. The consistent finding of increased IGF-1 SDS with LAGH warrants further investigation. Future research should prioritize long-term comparative studies, incorporate standardized and comprehensive QoL and adherence assessments, and further investigate the implications of differential IGF-1 responses across specific LAGH formulations and patient subgroups.

## Data Availability

The original contributions presented in the study are included in the article/**Supplementary Material**. Further inquiries can be directed to the corresponding author.
