# Supplementary material for: Weekly long-acting growth hormone versus daily growth hormone therapy on quality of life, efficacy, and safety in children with short stature: a systematic review and meta-analysis
Source: Front Endocrinol (Lausanne). 2026 Sep 2;17:1907184. doi: 10.3389/fendo.2026.1907184 (PMC13580145; doi:10.3389/fendo.2026.1907184)
Supplement: Supplementary file 1 [file SupplementaryFile1.doc]

**PubMed:**

#1 (long-acting OR "long acting" OR weekly OR once-weekly) AND (growth hormone OR gh OR hgh OR rhgh OR somatropin OR somatotropin)

#2 LAGH

#3 Ngenla

#4 somatrogon

#5 SKYTROFA

#6 Lonapegsomatropin

#7 Sogroya

#8 somapacitan

#9 #1 OR #2 OR #3 OR #4 OR #5 OR #6 OR #7 OR #8

#10 short stature OR dwarfism OR growth disorder* OR (growth AND disorder*) OR (growth AND deficiency)

#11 child* OR pediatric OR paediatric OR adolescent* OR infant*

#12 #10 AND #11

#13 #9 AND #12

**EmBase:**

#1 (long-acting or "long acting" or weekly or once-weekly).mp. [mp=title, abstract, heading word, drug trade name, original title, device manufacturer, drug manufacturer, device trade name, keyword]

#2 (growth hormone or gh or hgh or rhgh or somatropin or somatotropin).mp.

#3 #1 and #2

#4 LAGH or Ngenla or somatrogon or SKYTROFA or Lonapegsomatropin or Sogroya or somapacitan.mp.

#5 #3 or #4

#6 exp short stature/ or exp growth disorder/

#7 (short stature or dwarfism or growth disorder$).mp.

#8 #6 or #7

#9 exp child/ or exp adolescent/ or pediatric$.mp.

#10 #8 and #9

#11 #5 and #10

**Cochrane Library:**

#1 MeSH descriptor: [Human Growth Hormone] explode all trees

#2 (growth hormone OR gh OR hgh OR rhgh OR somatropin OR somatotropin):ti,ab,kw

#3 #1 OR #2

#4 (long-acting OR "long acting" OR weekly OR once-weekly):ti,ab,kw

#5 #3 AND #4

#6 LAGH OR Ngenla OR somatrogon OR SKYTROFA OR Lonapegsomatropin OR Sogroya OR somapacitan:ti,ab,kw

#7 #5 OR #6

#8 MeSH descriptor: [Dwarfism] explode all trees

#9 MeSH descriptor: [Growth Disorders] explode all trees

#10 (short stature OR dwarfism OR growth disorder*):ti,ab,kw

#11 #8 OR #9 OR #10

#12 MeSH descriptor: [Child] explode all trees

#13 (child* OR pediatric OR paediatric OR adolescent* OR infant*):ti,ab,kw

#14 #12 OR #13

#15 #11 AND #14

#16 #7 AND #15

**Web of Science:**

#1 TS=((long-acting OR "long acting" OR weekly OR once-weekly) AND (growth hormone OR gh OR hgh OR rhgh OR somatropin OR somatotropin))

#2 TS=(LAGH OR Ngenla OR somatrogon OR SKYTROFA OR Lonapegsomatropin OR Sogroya OR somapacitan)

#3 #1 OR #2

#4 TS=(short stature OR dwarfism OR growth disorder*)

#5 TS=(child* OR pediatric OR paediatric OR adolescent* OR infant*)

#6 #4 AND #5

#7 #3 AND #6
