# Supplementary material for: Weekly long-acting growth hormone versus daily growth hormone therapy on quality of life, efficacy, and safety in children with short stature: a systematic review and meta-analysis
Source: Front Endocrinol (Lausanne). 2026 Sep 2;17:1907184. doi: 10.3389/fendo.2026.1907184 (PMC13580145; doi:10.3389/fendo.2026.1907184)
Supplement: Supplementary file 2 [file SupplementaryFile2.doc]

Table S1. Risk of bias for included trial

| Trial | Random sequence generation | Allocation concealment | Blinding of participants and personnel | Blinding of outcome assessment | Incomplete outcome data | Selective reporting | Other bias |
| --- | --- | --- | --- | --- | --- | --- | --- |
| Peter 2012 [32] | Low | Unclear | High | Low | Low | Unclear | Unclear |
| Hwang 2013 [33] | Low | Unclear | High | Low | Low | Unclear | Unclear |
| Khadilkar 2014 [34] | Low | Unclear | High | Low | Low | Low | Low |
| Luo 2017 [35] | Low | Low | High | Low | High | Low | Low |
| Chatelain 2017 [36] | Low | Unclear | High | Low | Low | Low | Low |
| Zelinska 2017 [37] | Low | Unclear | High | Low | Low | Low | Low |
| Hwang 2018 [38] | Low | Unclear | High | Low | Low | Unclear | Unclear |
| Malievskiy 2018 [39] | Low | Unclear | High | Low | Low | Low | Unclear |
| Savendahl 2020 [41,42] | Low | Low | High | Low | Low | Low | Low |
| Sun 2021 [43] | Low | Unclear | High | Low | Low | Low | Low |
| Thornton 2021 [44] | Low | Unclear | High | Low | Low | Low | Low |
| Deal 2022 [46-48] | Low | Low | High | Low | Low | Low | Low |
| Horikawa 2022 [49] | Low | Unclear | High | Low | Low | Low | Unclear |
| Du 2022 [50] | Low | Unclear | High | Low | High | Low | Unclear |
| Mori 2024 [52] | Low | Low | High | Low | High | Low | Low |
| Liang 2025 [54] | Low | Low | High | Low | High | Low | Low |
| Fu 2025 [55] | Low | Low | High | Low | High | Low | Low |
| Abuzzahab 2026 [57] | Low | Low | High | Low | Low | Low | Low |
| Ying 2026 [58] | Low | Low | High | Low | High | Low | Low |
| Stawerska 2026 [60] | Low | Low | High | Low | High | Low | Low |

Table S2. Quality scores of prospective cohort studies using Newcastle-Ottawa Scale.

| Study | Selection | | | | Comparability | Outcome | | | NOS |
| --- | --- | --- | --- | --- | --- | --- | --- | --- | --- |
|  | Representativeness of the exposed cohort | Selection of the non exposed cohort | Ascertainment  of exposure | Demonstration that outcomes was not present at start of study | Comparability on the basis of the design or analysis | Assessment of outcome | Adequate follow-up duration | Adequate follow-up rate | Overall score |
| Qiao 2019 [40] | 1 | 1 | 1 | 1 | 2 | 1 | 1 | 1 | 9 |
| Wan 2021 [45] | 0 | 1 | 1 | 1 | 1 | 1 | 1 | 1 | 7 |
| Gao 2022 [51] | 0 | 1 | 1 | 1 | 1 | 1 | 1 | 1 | 7 |
| Xie 2024 [53] | 0 | 1 | 1 | 1 | 1 | 1 | 1 | 1 | 7 |
| Cai 2025 [56] | 1 | 1 | 1 | 1 | 2 | 1 | 1 | 0 | 8 |
| Guo 2026 [59] | 1 | 1 | 1 | 1 | 2 | 1 | 1 | 0 | 8 |
| Moutafi 2026 [61] | 0 | 1 | 1 | 1 | 1 | 1 | 1 | 1 | 7 |
