# Supplementary material for: Weekly long-acting growth hormone versus daily growth hormone therapy on quality of life, efficacy, and safety in children with short stature: a systematic review and meta-analysis
Source: Front Endocrinol (Lausanne). 2026 Sep 2;17:1907184. doi: 10.3389/fendo.2026.1907184 (PMC13580145; doi:10.3389/fendo.2026.1907184)
Supplement: Supplementary file 3 [file SupplementaryFile3.doc]

Figure S1. Sensitivity analysis for height velocity

Figure S2. Sensitivity analysis for height SDS

Figure S3. Sensitivity analysis for BA/CA

Figure S4. Sensitivity analysis for IGF-1 SDS

Figure S5. Sensitivity analysis for adverse events
