# Supplementary material for: Weekly long-acting growth hormone versus daily growth hormone therapy on quality of life, efficacy, and safety in children with short stature: a systematic review and meta-analysis
Source: Front Endocrinol (Lausanne). 2026 Sep 2;17:1907184. doi: 10.3389/fendo.2026.1907184 (PMC13580145; doi:10.3389/fendo.2026.1907184)
Supplement: Supplementary file 4 [file SupplementaryFile4.doc]

Figure S1. Funnel plot for height velocity

Figure S2. Trim and fill adjusted for height velocity

Figure S3. Funnel plot for height SDS

Figure S4. Funnel plot for BA/CA

Figure S5. Funnel plot for IGF-1 SDS

Figure S5. Funnel plot for adverse events
